# Contributions to Pathology

**Published:** 1831

**Authors:** 


### LVIII.

### CONTRIBUTIONS TO PATHOLOGY. By Dr. BARON.

DR. B. continues to supply matter for the pages of our respected Midland Cotemporary, and we wish he would appear more frequently on the stage, as he is not a routine physician, who studies only the means of filling his purse and extending his connexions. He is a zealous, though perhaps sometimes a little too theoretical pathologist.

#### I. *Abdominal Tumour.*

"The first case I shall mention, is that of a woman about fifty years of age. The disorder, as is generally the case, was not discovered till it had made some progress. It was then found, on examination, that several well-defined circular bodies occupied the lower part of the abdomen. Notwithstanding every effort to arrest their progress, the growth was rapid, and they ultimately acquired a magnitude greater in proportion to the rest of the body, than any tumour I have heard of. From the commencement, it was very clearly made out that it was composed of cysts of different sizes. Some of these cysts it was necessary to tap more than once, in order to relieve the patient from the painful effects of the increasing distention.

I may here mention an incident, connected with the passing of the trochar after death, in order to facilitate the dissection, which, had it occurred previously, would have been followed by the most afflicting consequences. One of the large cysts seemed to occupy the right side of the abdomen. It was thought advisable to empty this in the first instance. The instrument was, therefore, pushed in, but no fluid followed. The cause of this was clearly explained on pursuing the examination. The adventitious growth was so enormous, that it pressed up the diaphragm and the ribs, and forced the stomach out of its natural situation into the right side, its great curvature extending in a line from the margin of the ribs towards the ilium. In this situation the instrument was inserted. It transfixed the empty stomach, and penetrated the diseased mass beyond. It would have been truly frightful to have seen the poor creature's sufferings brought to an end by such an operation as this, had it been performed, as it really might have been, during her life-time. She had more than once been tapped on the opposite side; and no one could have anticipated such an occurrence as was met with in the other.

A crucial incision was next made through the integuments and muscles. The tumour was found very generally adherent to the peritoneum. After this adhesion had been partly separated, a cyst of considerable size, which manifestly contained fluid, was discerned. It was punctured, and about thirty pounds of a chocolate-coloured fluid were withdrawn. This was the cyst that had been repeatedly punctured during her life. On continuing the investigation a little farther, the displacement of the stomach was discovered, and, at the same time, the point where it was transfixed by the trochar. After some little difficulty, nearly the entire of the disorganization was removed. Its weight, independently of the fluid previously abstracted, was more than sixty pounds; so that the full weight must have been more than ninety pounds.

This immense mass was made up of a congeries of hydatids or vessels, or cysts of different magnitudes, and in different stages of their progress. The smallest were not larger than a garden pea, while the largest was of such capacity as to contain nearly 30lbs. weight of fluid. A great number were perfectly pellucid, and contained a thin watery fluid. Some of them, likewise, enclosed smaller cysts. These latter were in some instances consolidated, being nearly of the density of cartilage. In others, the cyst or sac was thickened and opake; the contents being thick and glairy in some instances; in others, yellow or purulent-looking; in others, the cavity had a cellular appearance, and was filled with a gelatinous-looking substance. In another portion, where the vesicles or cysts had grown together and pressed upon each other, the original globular form was lost, and the section of the diseased mass shewed all the varieties above-mentioned, but in a more advanced stage. The aggregation of the elementary parts, in some places, was so complete, and the approach towards consolidation was so great, that a complete scirrhus was nearly formed. The progress towards this state was quite discernible, the septa being formed by the original boundaries of the separate cysts, pressed and squeezed together by their respective growth, and giving a varied aspect to the morbid part.

The manner of the growth of this huge tumour was distinctly proved by its history. A few separate cysts were at first formed. Their boundaries could even be traced through the parietes of the abdomen. As they increased in size they approximated and united. The consequence was, that *at last, the original* inequalities of the surface could not be detected through the parietes, and a superficial examiner, on seeing the tumour removed from the abdomen, might imagine that the whole was enclosed in one sac or cyst. This fact illustrates a statement which I have often made, by showing how the boundaries of parts, originally distinct or separate from each other, may be lost as the disease advances."*

We quite agree with Dr. Baron, that the common idea of connecting the formation of all morbid growths with the process of inflammation, is an erroneous, and often a dangerous one. "There are still (says he) individuals (we would add, whole classes of individuals) who would place such an enormous adventitious growth as that as the above, as a consequence of inflammation." Yet cysts, in their primary condition, will be found in the centre of a viscus, or on the surface of a membrane, the surrounding parts being as free from all trace of disease as if no such bodies existed. There will be neither pain, redness, fever, nor any functional derangement in the body—circumstances which invariably attend inflammatory action, especially when it proceeds so far as to disorganize structures in the living machine.

"You shall see a cyst as large as an egg, for example, imbedded in the liver, with its transparent coats and pellucid contents. The viscus in which it is found, is, in no other respect, altered. Its colour is not changed, its investing membrane is as smooth and flexible as

* Midland Med. and Surg. Journal, No. XII.

ever, and the proper glandular texture will be found perfect up to the point of closest contact with this new formation. Is such a condition of things, let me ask, compatible with the existence of inflammation of any kind or degree? I am well aware that such occurrences are not often met with in man; and for this simple reason: death seldom takes place whilst these adventitious bodies are in their primary stage, and, when they proceed so far as to lead to death, they, as well as the surrounding parts, are in a very different state from what they were in their more early stages, at their commencement."

*Case* 2. The second case belonged to the same family, but the termination was unusual, and on that account, as well as from the instructive character of the morbid appearances, has been thought worthy of detail.

"The patient was a woman about thirty-seven years of age. She was admitted into the infirmary on the 30th of last Sept. The character of the disorganization under which she laboured, was easily discovered. The lower part of the abdomen was occupied by a tumour of considerable size. On passing the hand over it, an inequality was detected, both as regarded its surface and its consistence. That portion of it which occupied the right iliac region, was hard, and well-defined, and immovable; that which lay on the left side was soft, more diffused, and afforded a distinct sense of fluctuation. There was great tenderness over the surface of this part of the tumour, and frequently distressing dysuria.

There are great varieties in the rapidity of the growth of disorganizations of this kind. In the present instance, three years, according to the account of the patient, were required to bring it to its present size; during the last six months it had advanced more than in all the former period. The bowels act with difficulty; but she affirms that the *catamenia are regular*.

I need not trouble your readers with regular details of the treatment of this case, but shall state generally the remedies employed, and the result.—Leeches to the anus, and to the painful part of the abdomen, were repeatedly applied. The hydriodate of potash was used both externally and internally, and the bowels were kept open by saline aperients. These remedies were employed steadily for a month; the result was a diminution of the tenderness of the abdomen, and of the dysuria. The swelling on the right side, likewise, became softer and more moveable. On the 30th of October, the internal use of the hydriodate of potash was discontinued, and a solution of the chloride of potass was substituted. Under this treatment the general health improved, the swelling lessened, and both the bladder and the bowels acted with greater regularity. About the 13th of November, the pain in the tumour returned, and was followed by a considerable increase of size. Repeated applications of leeches were necessary, and the other remedies were continued till the 7th of December. The strong mercurial ointment was then substituted for the iodine ointment, and the tincture of iodine for the chloride of potass. By these means the tumour was again diminished in size, and the local distress much mitigated; so much so, that she asked leave to go home on urgent private affairs. She was absent from the infirmary for nearly three weeks, having returned on the 8th of January.

On the 14th she was seized with rigors, followed by heat of skin; then came on nausea, and frequent discharges from the bowels of a dirty greenish-coloured fluid. There was, at the same time, a marked reduction in the size of the abdominal tumour; but increased tenderness over its surface. Leeches and fomentations were applied to it, but without much benefit. Next day it was manifest that the whole surface of the peritoneum was affected; there was great constitutional irritation; rapid pulse; foul tongue; great prostration of strength, and all the symptoms of an alarming disease. She was bled at the arm; a blister was applied to the epigastrium; and a draught, containing liquor opii, manna, and tartrite of soda, was given every six hours. These remedies relieved the soreness and the irritation of the bowels; but on the 19th, she was seized with sickness, and copious vomiting of a dark green fluid, very much like that which had been passed per anum. After these last symptoms occurred, the patient only lived about two days. Before her death, the abdomen became again considerably tumefied.

The events which have been detailed, led clearly to a knowledge of some of the changes that had taken place. The discharge of the fluid from the anus; the shrinking of the abdominal tumour, and the subsequent vomiting, convinced me that one of the cysts had given way into the intestine. Such occurrences I have repeatedly met before, but death had, in no instance, followed. The cause of the mortality, in the present instance, will be sufficiently explained by the account of the morbid appearances.

On examining the body, the abdomen was found much distended. After cutting through its parietes, a large quantity of a dark fluid, having a very peculiar smell, and mixed with flakes of coagulated lymph, escaped. On throwing back the flaps made by the incision, several large cysts came into view, which occupied the lower part of the abdomen and the pelvis. The peritoneum was inflamed, and adhered, in several places, to the surface of the cysts. The intestines were forced upwards by the cysts, and adhered both to the latter and to each other. The bladder was likewise found high above the pubes, and expanded and flattened in such a manner by the pressure of the cysts, as to cover a considerable portion of their lower surface; so much so, indeed, that had any attempt been made to perforate either of the cysts at any point within two inches of the pubes, the bladder must have been transfixed. From circumstances unnecessary to mention, the examination was obliged to be performed in a hurried manner. In consequence of this, the most important morbid parts were quickly removed from the body: the opening, therefore by which the contents of one of the cysts had passed into the alimentary canal, was not satisfactorily made out. There was a very close and strong adhesion between one cyst and the rectum; but it was not at that point that the communication took place. This portion of the rectum, with the uterus and its appendages, the bladder and the whole of the adventitious growth, were then removed. This growth consisted of two large cysts; some smaller ones; and several solid tumours of different sizes. The parietes of the larger cysts were, in some places, very thin; in other places they had become very firm, and were three-quarters of an inch in thickness. In the posterior portion of that one which occupied the right iliac region, there was an ulcerated opening, more than half-an-inch in diameter, which permitted its contents to escape into the cavity of the abdomen; the character of the fluid in both cavities being alike.

Attached to the posterior part of the left cyst, were two smaller ones, each having thin and transparent coats; and the contents clear like water. There was another cyst adjoining these, about the size of a cricket-ball. The contents were of a dark brown colour; and the coats were thickened and changed like those of the larger ones. In the fundus of the uterus there was a small tumour in a scirrhous state, about the size of a horse-bean; and not far from it there was another, as large as a pigeon's egg, in a similar state. Both fallopian tubes were thickened and enlarged, so as to admit, with ease, a full-sized urethra bougie. The remains of the left ovary were found attached to one of the cysts, but the right ovary was so much enveloped in the diseased mass, that it could not be distinctly traced."

*Case* 3. Instances similar to that now to be detailed, are not very common. It would have been more complete had Dr. Baron been permitted to watch the disease to its termination. The patient, however, insisted on being removed from the hospital before death took place.

The patient was a female, aged 30 years, admitted into the Gloucester Infirmary on the 11th of November, 1830. An abstract of the symptoms and treatment is as follows :—

"The most prominent symptom, then, was the frequent discharge by the mouth, of a considerable quantity of a yellowish purulent-looking matter, occasionally mixed with blood, or, as she herself termed it, of *bloody corruption*. This discharge was generally preceded by a sense of sickness; the matter then ascended into the mouth, and was spit out. She described the action as that of vomiting; but to a by-stander it had not that character. There was no retching—there was no mucus, nor on any occasion were any of the usual contents of the stomach ever seen. These symptoms were accompanied by pain in the right side of the chest, between the shoulders, and in the right hypochondrium. Pressure on this part, and on the epigastrium, increased the pain, and a decided sense of hardness and fulness was at the same time felt. The woman looked ill, and was much emaciated. She could not lie on the right side; but she positively affirmed that she had no cough, and never had any, during any part of her illness.

Her disorder began about eight months ago, with severe pain in the right side, and a sense of weight in the epigastrium. Two months after the commencement of these pains, while engaged in some hard work, she brought up a considerable quantity of blood, which was soon afterwards followed by the *bloody corruption*. This continued daily for a fortnight; and she has never passed a week since, without its occurrence.

The circumstances connected with this discharge, rendered it somewhat difficult to account for its origin. The woman's own notion that it came through the stomach, was, in some degree, countenanced by the entire absence of cough, by the previous history of the disease, and the comparative freedom of the organs of respiration. The sound on the right side of the chest, it is true, was dull, but the respiratory murmur was tolerably complete.

After she had been a few days in the house, I ascertained, after a very accurate examination, that pressure on the right hypochondrium first occasioned a considerable degree of pain, and next, that sort of sensation which generally preceded the discharge. I immediately repeated the pressure, and continued it more firmly and steadily for a short time; and the result was, the almost immediate discharge of about two ounces of purulent matter from the mouth. It was manifestly forced up by the pressure; and I had an opportunity of verifying this fact on subsequent occasions. It was further ascertained that the alvine evacuations were of a very unnatural appearance; the food being undigested, and the bile manifestly deficient, but at no time was any trace of a purulent discharge detected in the stools.

From a careful review of all these occurrences, I was led to conclude that the abscess existed in the liver; but the *course* of the discharge was still somewhat perplexing. As it never passed *per anum;* as it never was mingled with any of the contents of the stomach; and as the *action* by which it was evacuated was not like that of vomiting, especially when the discharge was effected by pressure, the only conclusion I could come to was, that adhesion had taken place between the liver and the diaphragm, and between the latter and the pleura pulmonalis. Ulceration having subsequently destroyed the intervening portion of the diaphragm, the matter would easily escape into the lungs, and be discharged through the trachea. The absence of cough certainly militated against this view; I, nevertheless, saw no other method of accounting for the symptoms."

The subsequent details are very brief. Sometimes the patient seemed to improve considerably; but these flattering symptoms did not long continue. She began to have more serious affection of the chest. Pain was increased on full inspiration, and felt in both sides. On the 15th of December, and for the first time, she was seized with cough, followed by a discharge of purulent matter. All the phenomena now became alarming. The pulse increased in velocity — the emaciation was more marked — and each successive day announced the fatal termination, or at least the augmented progress of the disease. The unfortunate patient, under these circumstances, determined to be carried to her own home, there to end her miserable days. No dissection was permitted.

We hope Dr. Baron will not relax in his zealous pursuits in this department of pathological investigation.

